# Penalties for Non-Payment of Arrears

**Published:** 1914-02-14

**Authors:** 


### NATIONAL INSURANCE ACT DEVELOPMENTS.

### Penalties for Non-Payment of Arrears.

As indicated in our issue last week, important changes have recently taken place in the provisions of the National Insurance Act of 1911 regarding the payment of arrears of contributions. We there dealt with the concession granted to employed contributors of paying up back contributions, the non-payment of which was due to a period of unemployment, at a reduced rate. We shall now discuss another aspect of the question which is just as vital to the interests of insured persons—namely, what will be their position if, in spite of the abovementioned concession, the arrears of contribution are not paid.

The matter is now governed by Section 8 of the Amendment Act of 1913, which section entirely repeals the provisions of the principal Act regarding the penalties attaching to the non-payment of arrears. The alteration was made because under the original plan it was necessary for the average arrears to be worked out, and it was found that there were serious practical difficulties in arriving at a satisfactory way of calculating an average. Furthermore, except for the alteration of the system now inaugurated, it would have been impossible, in the opinion of the Insurance Commissioners, to permit the substitution of half-yearly insurance cards for the quarterly cards previously in vogue. Thus, in a very real sense, the new arrangements conduce to the simplification of the administrative machinery. No definite rules to be followed in the future are laid down by the section, and the drafting of suitable regulations is left entirely in the hands of the Insurance Commissioners. The wisdom of this course is undeniable, as the matter is one that requires most careful, expert handling, and this is much more likely to result by being left to the Insurance Commissioners and their advisers than if a cut-and-dried scheme had been laid down by Parliament in the Act itself. There is, moreover, a considerable gain in elasticity by the present arrangement.

### New Draft Regulations.

The Commissioners have not yet definitely adopted the new regulations that are about to be issued, but a scheme has been drawn up and published which appears likely to obtain their ultimate sanction. In order that the scheme might be fully discussed by experts the most representative gathering that has yet been held was convened in London on January 20 last. This meeting was a joint gathering of the Insurance Advisory Committees, and the members were unanimous in approving the draft of the scheme that was submitted to them. There is every reason, therefore, to suppose that they will ultimately be adopted by the Commissioners, and, in the circumstances, it will be interesting to consider the new proposals. The new rules differ from the old both in principle and in detail. It has already been remarked above that under the principal Act the penalties for non-payment of arrears were based upon the *average* number of weeks' contributions that had not been paid since entry into insurance.

Under that scheme also no reduction of benefit would have taken place until an average of four weeks' arrears per annum had been attained. The effect of this was to allow arrears to the extent of three weeks per annum on the average to be incurred without penalty. By this means also contributors who had been insured for a long period were allowed a larger total number of contributions in arrear before being liable to reduction of benefit than persons insured for a short while only. These two features are preserved by the new draft regulations, although the proposed method of carrying them into effect is changed.

### The Scheme in Outline.

The general plan of the draft regulations is as follows: At the end of each contribution year (ending in June) the total arrears of each member for the year are to be ascertained. In this calculation no account will be taken of contributions that were unpaid owing to the sickness of the employed contributor. In this respect, as also with regard to contributions which were allowed to fall into arrear during the first year of the operation of the Act, no alteration has been made. "Each member is then credited in the contribution register with his reserve contributions—*i.e.* three contributions for a completed year, with a proportionate number where the member has been insured for less than a year. He is also credited with any reserve contributions brought forward from the previous year, and with any arrears paid off during the year."

If on balance he is found to be still in arrear, there will be sent to him, with his insurance book, an arrears card, by means of which he will be informed of his position, and be enabled to see what payment he must make if he wishes to remain entitled to benefits at the full scale. An illustration may perhaps make these regulations rather more clear. Let us suppose that a man first became insured on July 1, 1913, and that during the year July 1913 to July 1914 he is unemployed for two weeks, and the contributions unpaid are allowed to fall into arrear. Then shortly after July 1, 1914, the contribution register will be made up, and he will stand thus: Gross arrears, two weeks; reserve contributions to be credited, three weeks; leaving a balance in his favour of one week. Now let us assume that during the year July 1914 to July 1915 he is unemployed for five weeks, and does not himself pay the contributions. His position after July 1, 1915, will then be: Gross arrears, five weeks; reserve contributions for current year, three weeks; reserve contributions brought forward from previous year, one week. He will thus have a credit of four weeks against gross arrears of five weeks, leaving one week in arrear on balance. This balance will form the basis for the penalties.

It is, of course, open to the insured person to pay off arrears at any time during the contribution year in which they were incurred, and it will be to his distinct advantage to do so in the early years of insurance, as by this means he will accumulate quite a large number of reserve contributions to be carried forward. Thus, if a person has been insured for ten years and has kept up his contributions to the full number of fifty-two each year, excluding any weeks for which he was in receipt of sickness or disablement benefits, he will, at the end of that time, have thirty weeks' reserve contributions to his credit, and will therefore not incur penalty at the end of the year unless in that year he has been out of employment for thirty weeks and has not paid the contributions falling due during that period.

When the net arrears at the end of any contribution year have been ascertained in the foregoing manner, it is proposed that the employed contributor shall be given until October 1 to pay off the arrears by affixing the appropriate stamps on an arrears card. If, however, he does not take advantage of these days of grace, he will, during the twelve months commencing on the following November 1, suffer certain penalties.

### The Penalties.

The suggested penalties are as follows:—

(*a*) If the penalty arrears do not exceed sixteen the sickness benefit will be reduced during the first six weeks of sickness by 7d. a week (6d. in the case of women) for each penalty arrear, provided that the sickness benefit as thus reduced will not be less than 2s. a week (1s. 6d. a week in the case of a woman).

(*b*) If the penalty arrears exceed sixteen but do not exceed twenty he will be suspended from benefit for the first six weeks of sickness during that year.

(*c*) If the penalty arrears exceed twenty but do not exceed twenty-six all sickness and disablement benefits will be suspended for one year.

(*d*) If the penalty arrears exceed twenty-six all benefits will be suspended for one year.

It will be seen that the main idea of the proposals is to make each member pay his arrears as he goes along, either in actual cash or by suffering a reduction of benefit, and that arrears incurred during any one contribution year are in any case entirely wiped out sixteen months after the close of that year. A close investigation of the regulations reveals the fact that in certain circumstances somewhat anomalous positions may arise. For instance, a man who is seventeen weeks in arrear on balance obtains no sickness benefit at all during the first six weeks of sickness if such occur during the following benefit year. A man sixteen weeks in arrear is, however, entitled to 2s. a week for a corresponding period. It would therefore seem that for a payment of 4d., if made before the appropriate October 1, the contributor would secure for himself the right to receive 2s. a week for the first six weeks of sickness. This is an anomaly due to the scale, and we imagine that few will be found who would not exercise the option of paying 4d. Moreover, if a person is, on balance, over twenty-six weeks in arrear, and cannot afford to pay the number of contributions necessary to entitle him to any benefit, he would be well advised not to make any payment at all, as his position at the end of the following year would be unaffected by any such payment.

### A Valuable Option.

It may also be pointed out that the new proposals give to the employed contributor an option which is of considerable value to him, and which may be used to the detriment of his approved society unless due allowance has been made for it in the calculation of the penalties. It has already been remarked that if at the end of a particular "contribution year" a member is sufficiently in arrear to incur a penalty, he may either pay off the sum that is due in cash and thus be eligible for full benefits, or he may elect instead to suffer a reduction of benefit should he fall ill during the ensuing "benefit year." If such a member is of a robust nature, and usually enjoys good health, he will most likely elect not to pay in cash but to take his chance of falling ill. If, on the other hand, he is of a delicate constitution, he will prefer, if possible, to qualify for full benefits. In either case, therefore, the approved society will stand to suffer loss through the option being exercised against it.

Apart from these more or less minor defects, however, the new regulations appear to be a considerable improvement on the old. Their principal virtue is that of comparative simplicity, and in this respect they are a great advance upon the original scheme. It may be pointed out that the regulations will probably come into operation in July next, and certainly until then no reduction or suspension of benefit on account of arrears will take place.

